# Radiological Reporting of Brain Atrophy in MRI: Real-Life Comparison Between Narrative Reports, Semiquantitative Scales and Automated Software-Based Volumetry

**DOI:** 10.3390/diagnostics15101246

**Published:** 2025-05-14

**Authors:** Federico Bruno, Cristina Fagotti, Gaspare Saltarelli, Giovanni Di Cerbo, Alessandra Sabatelli, Claudia De Felici, Antonio Innocenzi, Ernesto Di Cesare, Alessandra Splendiani

**Affiliations:** 1 Department of Biotechnological and Applied Clinical Sciences, University of L’Aquila, 67100 L’Aquila, Italy; 2 Neuroradiology, San Salvatore Hospital, 67100 L’Aquila, Italy

**Keywords:** MRI, dementia, brain atrophy, brain volumetry, automated software

## Abstract

**Background**: Accurate assessment of brain atrophy is essential in the diagnosis and monitoring of brain aging and neurodegenerative disorders. Radiological methods range from narrative reporting to semi-quantitative visual rating scales (VRSs) and fully automated volumetric software. However, their integration and consistency in clinical practice remain limited. **Methods**: In this retrospective study, brain MRI images of 43 patients were evaluated. Brain atrophy was assessed by extrapolating findings from narrative radiology reports, three validated VRSs (MTA, Koedam, Pasquier), and Pixyl.Neuro.BV, a commercially available volumetric software platform. Agreement between methods was assessed using intraclass correlation coefficients (ICCs), Cohen’s kappa, Spearman’s correlation, and McNemar tests. **Results**: Moderate correlation was found between narrative reports and VRSs (ρ = 0.55–0.69), but categorical agreement was limited (kappa = 0.21–0.30). Visual scales underestimated atrophy relative to software (mean scores: VRSs = 0.196; software = 0.279), while reports tended to overestimate. Agreement between VRSs and software was poor (kappa = 0.14–0.33), though MTA showed a significant correlation with hippocampal volume. Agreement between reports and software was lowest for global atrophy. **Conclusions**: Narrative reports, while common in practice, show low consistency with structured scales and quantitative software, especially in subtle cases. VRSs improve standardization but remain subjective and less sensitive. Integrating structured scales and volumetric tools into clinical workflows may enhance diagnostic accuracy and consistency in dementia imaging.

## 1. Introduction

Brain atrophy is a hallmark of many neurodegenerative diseases and plays a pivotal role in understanding their underlying pathophysiology [1,2,3,4,5,6]. Magnetic resonance imaging (MRI) has become an essential tool for detecting and monitoring brain atrophy, providing detailed visualization of affected regions and enabling quantification of tissue loss. Structural MRI, in particular, is widely used to evaluate atrophy patterns associated with various forms of dementia, aiding both in diagnosis and longitudinal disease tracking. Imaging biomarkers—such as regional volume loss—are crucial for improving early detection and differential diagnosis of neurodegenerative conditions. Volumetric MRI, multiparametric approaches, and molecular imaging techniques have further enhanced diagnostic sensitivity and specificity [2,7,8,9,10,11,12].

MRI has become increasingly important in characterizing primary neurodegenerative pathology, not only by detecting regional brain atrophy but also by identifying other structural markers associated with aging and neurodegeneration. These include white matter hyperintensities (WMHs), cerebral microbleeds, and enlarged perivascular spaces, which provide valuable insights into underlying small vessel disease and overall brain health. The detection of these features enhances the diagnostic accuracy for various dementia subtypes and contributes to a more comprehensive assessment of brain aging [8,13,14,15,16,17,18].

Importantly, brain atrophy is not exclusive to pathology: it is also a feature of normal aging, which is typically associated with diffuse and gradual brain volume reduction. However, unlike the selective and region-specific patterns of neurodegeneration, age-related atrophy tends to occur more uniformly and progresses at a slower rate. Distinguishing between physiological tissue loss and pathological atrophy is particularly critical in the context of today’s aging population, where increasing life expectancy is contributing to a higher prevalence of cognitive decline and dementia-related conditions. Accurate differentiation is essential for timely diagnosis, appropriate clinical management, and avoiding both over- and underdiagnosis in older individuals [17,19,20,21,22].

To improve interpretability and consistency, semi-quantitative visual rating scales (VRSs) such as the Medial Temporal Atrophy (MTA) scale, Global Cortical Atrophy (GCA) Scale, and the Fazekas scale for white matter hyperintensities have been introduced. These tools enable structured evaluation of imaging findings, but their use in routine clinical practice remains variable [8,13,15,16].

The lack of standardization in radiological reporting presents a significant challenge in both clinical practice and research. Unstructured or free-text reports are still widely used, despite their inherent limitations. These reports rely heavily on subjective interpretation and may omit essential details required for a comprehensive diagnostic assessment. In contrast, structured reporting has been proposed to enhance standardization. Structured reports utilize predefined templates tailored to specific diseases or clinical indications, ensuring systematic inclusion of all relevant information [23].

In the context of neurodegenerative disorders, variability in reporting is particularly problematic. Critical findings, such as the presence and extent of brain atrophy or white matter hyperintensities, may be under-reported or inconsistently described. Furthermore, the use of semi-quantitative scales is often inconsistent or absent. This lack of standardization can make it difficult to compare results between centers and to integrate imaging data into large-scale studies aimed at understanding disease progression or treatment response [9,24].

The concept of “contextual reporting” has emerged as a compromise between structured and free-text reporting, where the structure of the report is tailored to the specific pathology being evaluated, ensuring consistent capture of essential details while allowing the radiologist the freedom to provide individualized insights [25,26,27]. Radiological rating scales (VRSs) play a crucial role in this area, allowing for better recognition of radiological findings in diagnostic protocols for dementia. The literature demonstrates that the characteristic findings of brain atrophy are often underdiagnosed and not consistently reported in radiological reports. It is suggested that the personal experience of the radiologist and the predominant use of imaging to exclude secondary and reversible causes of dementia are key factors underlying the discrepancies observed between reports from different hospitals. Furthermore, the use of the VRS remains poorly documented. At the European level, 75% of centers utilize the VRS in clinical practice, 82% report changes in white matter, and only 6% regularly employ quantitative volumetric measurements [8,13,15,16]. Among the most widely used scales, the Medial Temporal Atrophy (MTA) scale is the most prevalent. The main barrier to widespread use of the VRS is the lack of specific training required to ensure a high level of intra-rater agreement [13].

In parallel, with the exploding diffusion of AI-based solutions in neuroradiology, the use of dedicated software for automated brain volumetry is becoming increasingly common in clinical practice [28,29,30]. These tools offer objective, reproducible measurements of brain structures and have the potential to complement visual assessments, particularly in early or subtle cases of atrophy [31,32,33,34].

This study aims to evaluate how brain atrophy is currently documented in clinical MRI reports and to assess the consistency of these reports with findings derived from visual rating scales and automated volumetric software. Specifically, we investigate the degree of concordance between narrative radiology reports, semi-quantitative visual scales, and software-based metrics, with the goal of understanding the potential complementary role of automated tools in the diagnostic workflow for neurodegenerative disease.

## 2. Materials and Methods

### 2.1. Study Population

This observational study was conducted on a cohort of 43 patients (10 women, 33 men, with a mean age of 67.65 ± 9.53 years) retrospectively selected from a cohort previously enrolled in a prior study conducted at our institution [35].

All patients had been scanned using a standardized MRI protocol to ensure consistency in image acquisition and comparability across the various methods of analysis.

Inclusion criteria consisted of the availability of a 3D T1-weighted volumetric sequence suitable for automated processing, high-quality structural MR images and an absence of significant motion artifacts.

Patients were excluded if automated segmentation failed or was deemed technically inappropriate due to artifacts, incomplete sequences, or abnormal anatomy that interfered with the software’s algorithms.

### 2.2. MRI Acquisition Protocol

All MRI studies were performed on the same 3 Tesla scanner (MR750w, GE Healthcare, Chicago, IL, USA) using a 32-channel phased-array head coil. The standardized protocol included the following sequences:

Axial T2-weighted FLAIR: slice thickness 3.0 mm (gap 0.3 mm), TR 11,000 ms, frequency FoV 24 cm, phase FoV 0.8.

Axial T2 GRE*: slice thickness 3.0 mm (gap 0.3 mm), TR 960 ms, frequency FoV 26 cm, phase FoV 0.75.

Axial SWI: slice thickness 2.0 mm, frequency FoV 24 cm, phase FoV 0.85.

Axial DWI: slice thickness 3.0 mm (gap 0.3 mm), TR 10,550 ms, frequency FoV 26 cm, phase FoV 0.8.

Axial and coronal T2-weighted: slice thickness 3.0 mm (gap 0.3 mm), TR 7854 ms, frequency FoV 26 cm, phase FoV 0.8.

Volumetric T1-weighted 3D-IR-FSPGR: slice thickness 1 mm (no gap), TR 8.5 ms, frequency FoV 25.6 cm, phase FoV 0.8.

All image datasets were anonymized prior to analysis to ensure patient confidentiality in accordance with institutional and ethical guidelines.

### 2.3. Automated Brain Volumetry

Quantitative brain volumetry was conducted using Pixyl.Neuro, a CE-marked, commercially available software platform developed by Pixyl SA (Grenoble, France). This tool enables automated segmentation and analysis of brain volumes using high-resolution 3D T1-weighted images.

The software performs two main types of segmentation: tissue-level segmentation, which distinguishes gray matter (GM), white matter (WM), and cerebrospinal fluid (CSF), and structure-level segmentation, which identifies and quantifies specific brain structures including the hippocampi, thalami, putamen, and cortical lobes.

Following segmentation, the software computes the absolute volumes of these structures (in milliliters) and compares them to an internal normative database adjusted for patient age and sex. This yields percentile rankings, which express each structure’s volume relative to a healthy reference population.

**Figure 1:**
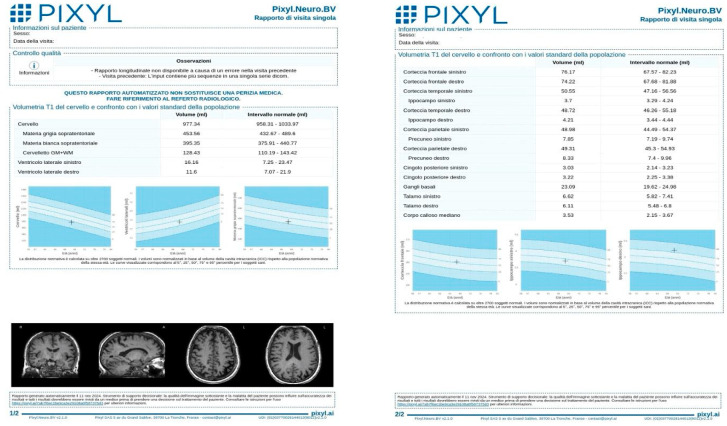
Example of Pixyl report after MRI brain volumetry, illustrating the software’s quantitative volumetric measurements of brain structures. The report includes measures of global brain volume, regional volumes (e.g., hippocampus, ventricles), and comparisons to an age-matched normative database.

An example of a Pixyl report is provided (Figure 1), illustrating the software’s output, including global brain volumes, regional metrics, and percentile comparisons.

### 2.4. Visual Rating Scales (VRSs)

Each patient’s MRI was independently evaluated by two trained neuroradiologists using three well-validated visual rating scales (VRSs) (Table 1).

Medial Temporal Atrophy (MTA) scale [36]: The MTA scale assesses atrophy in the medial temporal lobe structures, specifically focusing on the hippocampus, choroid fissure, and temporal horn of the lateral ventricle. Ratings range from 0 (no atrophy) to 4 (severe atrophy).

Scores ≥ 2 were considered abnormal for patients < 75 years; scores ≥ 3 were abnormal for patients ≥ 75 years.

Global Cortical Atrophy (GCA) Scale [37]: This scale evaluates sulcal widening and ventricular enlargement across all lobes. Each hemisphere is scored from 0 to 3, with higher scores indicating greater Global Cortical Atrophy. Scores of ≥2 were considered pathological in this study.

Posterior Atrophy scale of parietal atrophy [38]: Designed to assess parietal and posterior cingulate atrophy, this scale also uses a 0–3 range. A score of ≥2 was considered abnormal.

### 2.5. Qualitative Evaluation of Original Radiology Reports

Original narrative radiology reports corresponding to each MRI exam were reviewed independently to determine how the findings were described in routine clinical practice. Specifically, the reports were examined for any mention or description of medial temporal, global cortical, or posterior cortical atrophy—corresponding to the anatomical regions evaluated by the VRS.

A qualitative scoring system adapted from Torisson [4] was applied to classify how brain atrophy was documented in the original reports:

- NA: No mention of atrophy or related findings.
- 0: Atrophy explicitly reported as absent or normal.
- 1: Atrophy mentioned in vague or mild terms, without specific grading.
- 2: Atrophy described as moderate.
- 3: Atrophy described as severe.

This scoring allowed for a structured comparison between routine radiology practice (narrative reporting), visual rating by trained observers, and quantitative volumetric data generated by automated software.

### 2.6. Statistical Analysis

All statistical analyses were performed using MedCalc software version 23.1.6 (MedCalc Software Ltd., Ostend, Belgium). The primary objective of the analysis was to assess the degree of agreement and correlation between three different methods of brain atrophy assessment: narrative radiology reports, semi-quantitative visual rating scales, and automated volumetric analysis using Pixyl.Neuro.

Spearman’s rho (ρ) was calculated to evaluate the correlation between narrative radiological interpretations and established visual rating scales (VRSs), calculating the relationship between the Torrison classification and three VRSs: the Medial Temporal Atrophy (MTA) scale, the Koedam scale for posterior atrophy, and the Pasquier scale for Global Cortical Atrophy.

Agreement between categorical or ordinal variables was evaluated using Cohen’s kappa (κ) coefficient, while intraclass correlation coefficients (ICCs) were used to assess the consistency of continuous or quasi-continuous variables across methods. Kappa values were interpreted using standard benchmarks: <0.20 (slight), 0.21–0.40 (fair), 0.41–0.60 (moderate), 0.61–0.80 (substantial), and >0.80 (almost perfect agreement).

For each atrophy assessment method (report, VRS, software), concordance rates were calculated to determine how often the methods reached the same classification (normal vs. abnormal). Discrepancies were further analyzed descriptively to explore common patterns of under- or over-reporting.

A *p*-value < 0.05 was considered statistically significant for all comparisons.

## 3. Results

### 3.1. Report - VRS Comparison

Spearman rank correlation analysis demonstrated a moderate to strong positive correlation between the Torrison classification of narrative radiological reports and visual rating scales (VRSs). Specifically, correlation coefficients (ρ) were 0.56 for the Medial Temporal Atrophy (MTA) scale (*p* < 0.001), 0.69 for the Koedam scale (*p* < 0.001), and 0.55 for the Pasquier scale (*p* < 0.001),

For the Pasquier scale, the ICC for individual measures (i.e., comparing each case independently) was 0.54 with a 95% confidence interval ranging from 0.31 to 0.72. This indicates a moderate level of reliability between the visual scale and the radiology report. When assessing the average of the scores across observers or cases, the ICC increased to 0.71 (95% CI: 0.47–0.84), reflecting improved consistency. However, when using Cohen’s kappa, which looks at categorical agreement, the value was 0.21 (95% CI: 0.01–0.41), which corresponds to only modest agreement between the two reporting approaches.

A similar trend was observed with the Koedam scale. The ICC for single measures was 0.55 (95% CI: 0.078–0.78), again indicating a moderate level of consistency. The ICC for average measures was slightly higher at 0.71 (95% CI: 0.14–0.87). The Cohen’s kappa coefficient for the Koedam scale and narrative reports was 0.30 (95% CI: 0.14–0.46), indicating fair agreement.

When evaluating the MTA scale, the ICC for single scores was 0.47 (95% CI: 0.23–0.72), with the mean score ICC reaching 0.68 (95% CI: 0.18–0.78). This shows that, while there is some alignment between narrative reporting and standardized visual evaluation, the agreement is only moderate at best.

The ICC results are reported in Table 2.

### 3.2. VRS - Software Comparison

The mean score derived from the visual scales was 0.196, whereas the software yielded a higher average score of 0.279. This suggests that, on average, visual assessment tends to underestimate the degree of brain atrophy compared to the objective quantitative values generated by the software.

For the MTA scale, the kappa was 0.14, with 71.73% concordance and a statistically significant McNemar *p*-value of 0.005, suggesting a discrepancy in how often the software and scale classified the same cases as abnormal.

For the Koedam scale, the kappa was 0.33, with 82.61% concordance and a non-significant McNemar *p* = 0.28.

For the Pasquier scale, the kappa was 0.29, concordance was 80.43%, and the McNemar *p* = 0.18.

Concordance results between visual rating scales and software-based volumetric analysis are summarized in Table 3.

### 3.3. Report—Software Comparison

Looking at agreement between software and radiologist descriptions for temporal, global, and posterior brain regions, in the temporal region, the kappa was 0.29, with a concordance rate of 84.78%, and a McNemar *p* = 0.13.

For the global cortical region, agreement was markedly lower: kappa = 0.03, concordance = 69.56%, and McNemar *p* = 0.50.

In the posterior region, the kappa was 0.20, with 80.43% concordance and a McNemar *p* = 0.78.

It is important to note that the McNemar test values are non-significant across temporal (*p* = 0.13), global (*p* = 0.50), and posterior (*p* = 0.78) regions. This suggests that the discrepancies observed between the qualitative radiological reports and the quantitative software measurements may not be statistically significant.

Results of the concordance evaluation between radiology reports and automated volumetric measurements are summarized in Table 4.

To summarize the overall diagnostic patterns, we dichotomized the findings from each method into “normal” (0) and “pathological” (1) and directly compared the frequency of classifications across the three assessment modalities:

- Narrative radiology reports classified 17 cases as pathological and 26 as normal.
- Visual rating scales reported only 8 cases as pathological and 35 as normal.
- Software-based volumetry identified 12 cases as pathological and 31 as normal.

**Figure 2:**
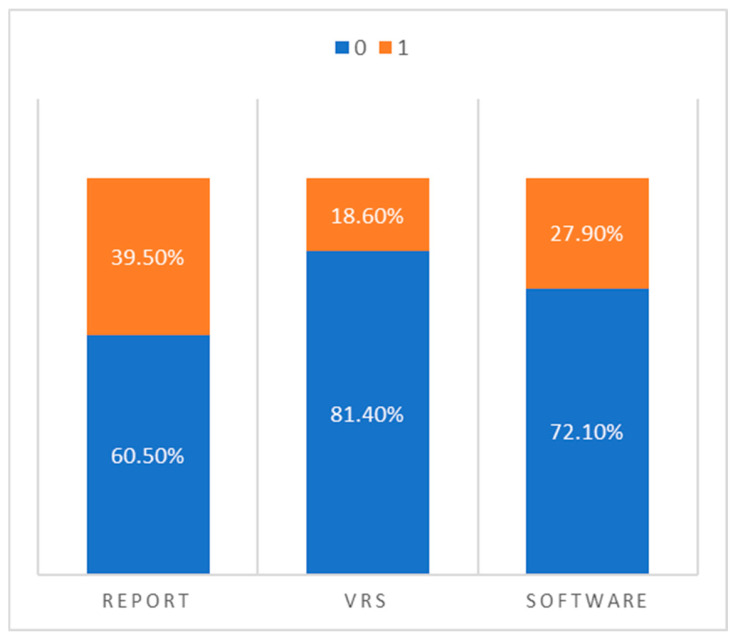
Percentage distribution of “normal” vs. “pathological” classifications across radiology reports, visual rating scales, and automated volumetric software.

This comparison, illustrated in Figure 2, highlights a key trend: visual rating scales appear to under-report pathological atrophy, while radiology reports tend to overestimate it compared to the more conservative and statistically grounded classifications provided by the software.

## 4. Discussion

Accurately detecting and characterizing brain atrophy is a central goal in the diagnostic work-up of brain aging, dementia syndromes and several neurodegenerative disorders. A variety of assessment tools exist—ranging from traditional narrative reports to visual rating scales (VRSs) and fully automated volumetric software—each with distinct advantages and limitations [39].

Our study compared these three approaches in a real-world cohort, revealing important discrepancies in classification, agreement, and reliability.

Narrative reports, while dominant in routine clinical practice, rely heavily on the subjective impression of the radiologist, informed by clinical context and visual inspection of MRI or CT scans. Despite their ubiquity, they are limited by low sensitivity and high inter-observer variability. As Harper et al. noted, narrative reporting often fails to detect subtle atrophic changes and is unsuitable for longitudinal monitoring, largely due to the absence of standardized descriptors. Even among experienced neuroradiologists, inter-rater agreement for identifying hippocampal atrophy or posterior cortical thinning has been shown to be poor [14,40].

In our study, although narrative reports moderately correlated with structured VRS assessments (ρ = 0.56–0.69), the agreement was only modest when categorized, as evidenced by low kappa values (0.21–0.30). This reflects the qualitative, variable nature of narrative descriptions and the lack of standard reporting frameworks in current practice. Furthermore, narrative reports tended to overestimate pathological atrophy relative to both VRS and volumetric analysis, likely reflecting an interpretive bias toward caution.

Semi-quantitative visual rating scales represent a compromise between subjective and quantitative methods. Commonly used tools include the Medial Temporal Atrophy (MTA), Global Cortical Atrophy (GCA), and Posterior Atrophy (Koedam) scales. These tools have been widely validated in dementia research and show moderate to good correlation with neuropathological and clinical severity markers. For example, Fumagalli et al. demonstrated that a visual scale specifically assessing parieto-occipital sulcus widening could reliably distinguish posterior cortical atrophy (PCA) from typical Alzheimer’s disease, highlighting the value of tailored scales in subtype differentiation [4,8,22].

Our results align with these observations. The MTA scale, in particular, showed a statistically significant association with hippocampal volumes from the Pixyl software and had the highest clinical consistency. However, agreement with software remained low across all scales when analyzed categorically (e.g., κ = 0.14–0.33), and VRSs consistently underestimated atrophy, especially in borderline cases. As Harper et al. also noted, while VRSs improve diagnostic consistency over narrative reporting, they are still affected by inter-rater variability and require considerable experience for reliable use [14].

Automated volumetric tools, such as commercially available AI based software, offer objective, reproducible brain volume assessments using normative databases adjusted for age and sex. This facilitates early detection of neurodegeneration and is particularly useful in ambiguous or early-stage cases. In line with our findings, Zilioli et al. reported that volumetric analysis better detected early hippocampal and parietal atrophy and more accurately predicted amyloid PET positivity than visual assessment in patients being evaluated for anti-amyloid therapy. They found automated tools to be especially valuable in identifying candidates for disease-modifying treatments [22].

Persson et al. also demonstrated a strong correlation (r = 0.79) between NeuroQuant-derived hippocampal volumes and MTA scores, with volumetry better differentiating MCI from early dementia. They emphasized that automated quantification adds diagnostic value in borderline presentations—supporting our own observation that narrative reports and VRSs diverge most notably from software results in mild or ambiguous cases [21].

Despite their promise, volumetric tools are not without limitations. One key concern is inter-software variability. As Pemberton et al. highlighted in a systematic review, different commercial software platforms may yield non-interchangeable results due to differences in segmentation algorithms, reference datasets, and preprocessing pipelines [41]. This variability complicates multicenter comparisons and reduces confidence in cross-software reproducibility. Our findings also reflect this criticism: while Pixyl provides useful normative comparisons, discrepancies with VRSs and narrative interpretations remain significant and unresolved.

Additionally, our study has limitations. The sample size was relatively small, and many patients had little or no measurable atrophy, limiting statistical power. It is plausible that agreement across methods would improve in cohorts with more advanced neurodegeneration. The Torisson system used to retrospectively score narrative reports is also not a validated clinical tool but provides a useful framework for qualitative-to-quantitative comparison. A key limitation of this study is the absence of correlation with clinical cognitive scales and other imaging modalities, which would be essential for comprehensive validation of imaging findings.

This study demonstrates that while narrative reports remain the standard in clinical neuroimaging, they frequently diverge from structured visual scales and automated software tools—especially in early or subtle presentations of brain atrophy. Visual rating scales offer better standardization but still may underestimate atrophy. Automated volumetric software provides greater sensitivity and reproducibility, especially in ambiguous cases, but suffers from tool-dependent variability that limits comparability.

Future efforts should focus on standardizing VRS use in clinical reports through training and templates, improving volumetric software harmonization across platforms, and developing integrated reporting models that combine qualitative and quantitative findings to enhance diagnostic confidence and accuracy.

## Figures and Tables

**Table 1:** A detailed summary of all three scales, including anatomical targets, imaging planes, scoring systems, and thresholds for pathological atrophy.

Scale	Region Assessed	Imaging Plane/Sequence	Scoring Range	Scoring Criteria	Pathological Cut-Off
**MTA (Scheltens)**	Medial temporal lobe (hippocampus, choroid fissure, temporal horn)	Coronal T1-weighted	0–4	0 = normal; 1 = mild choroid fissure widening; 2 = +mild temporal horn enlargement; 3 = +moderate hippocampal atrophy; 4 = severe atrophy with structural loss	≥2 (<75 yrs); ≥3 (≥75 yrs)
**GCA (Pasquier)**	Global Cortical Atrophy (frontal, parietal, temporal, occipital lobes)	Axial T1-weighted	0–3 (per hemisphere)	0 = normal; 1 = mild sulcal widening; 2 = moderate; 3 = severe “knife blade” atrophy	≥2 (any age)
**Koedam**	Posterior parietal regions (precuneus, parieto-occipital sulcus, posterior cingulate)	Axial, sagittal, coronal T1-weighted	0–3	0 = no sulcal widening; 1 = mild; 2 = moderate; 3 = severe widening and atrophy	≥2 (any age)

**Table 2:** Agreement between radiology reports and visual rating scales (VRSs): intraclass correlation coefficients.

Visual Rating Scale	ICC (Single Measures)	ICC (Average Measures)
Pasquier (GCA)	0.54 (95% CI: 0.31–0.72)	0.70 (95% CI: 0.47–0.83)
Koedam	0.55 (95% CI: 0.07–0.78)	0.71 (95% CI: 0.14–0.87)
MTA (Scheltens)	0.47 (95% CI: 0.23–0.72)	0.68 (95% CI: 0.18–0.78)

**Table 3:** Comparison of agreement between visual rating scales and the software-based volumetry.

VRS—Software	Cohen Kappa Test	Concordance	McNemar Test—*p* Value
MTA	0.14	71.73%	0.005
Koedam	0.33	82.61%	0.28
Pasquier	0.29	80.43%	0.18

**Table 4:** Comparison of the agreement between radiological reports and software-based volumetric analysis.

Report—Software	Cohen Kappa	Concordance	McNemar Test—*p* Value
Temporal	0.29	84.78%	0.13
Global	0.03	69.56%	0.50
Posterior	0.20	80.43%	0.78

## Data Availability

The datasets generated and analyzed for the current study are available from the corresponding author upon reasonable request.
